# Influenza Virus A/Beijing/501/2009(H1N1) NS1 Interacts with β-Tubulin and Induces Disruption of the Microtubule Network and Apoptosis on A549 Cells

**DOI:** 10.1371/journal.pone.0048340

**Published:** 2012-11-06

**Authors:** Xueqing Han, Zhihui Li, Hongjun Chen, Huiyu Wang, Lin Mei, Shaoqiang Wu, Tianyi Zhang, Bohua Liu, Xiangmei Lin

**Affiliations:** 1 Chinese Academy of Inspection and Quarantine, Beijing, China; 2 State Key Laboratory of Pathogens and Bio-security, Beijing Institute of Microbiology and Epidemiology, Beijing, China; 3 College of Veterinary Medicine, China Agricultural University, Bejing, China; Centre of Influenza Research, The University of Hong Kong, Hong Kong

## Abstract

NS1 of influenza A virus is a key multifunctional protein that plays various roles in regulating viral replication mechanisms, host innate/adaptive immune responses, and cellular signalling pathways. These functions rely on its ability to participate in a multitude of protein-protein and protein-RNA interactions. To gain further insight into the role of NS1, a tandem affinity purification (TAP) method was utilized to find unknown interaction partner of NS1. The protein complexes of NS1 and its interacting partner were purified from A549 cell using TAP-tagged NS1 as bait, and co-purified cellular factors were identified by mass spectrometry (MS). We identified cellular β-tubulin as a novel interaction partner of NS1. The RNA-binding domain of NS1 interacts with β-tubulin through its RNA-binding domain, as judged by a glutathione S-transferase (GST) pull-down assay with the GST-fused functional domains of NS1. Immunofluorescence analysis further revealed that NS1 with β-tubulin co-localized in the nucleus. In addition, the disruption of the microtubule network and apoptosis were also observed on NS1-transfected A549 cells. Our findings suggest that influenza A virus may utilize its NS1 protein to interact with cellular β-tubulin to further disrupt normal cell division and induce apoptosis. Future work will illustrate whether this interaction is uniquely specific to the 2009 pandemic H1N1 virus.

## Introduction

Influenza A viruses are globally important human and animal respiratory pathogens that are responsible for both seasonal, endemic outbreaks, and periodic unpredictable world-wide pandemics [1]. Three human pandemics occurred during the last century [2].The worst influenza A pandemic on record in 1918 killed approximately 50 million people worldwide [3]. In 2009, a novel swine-origin H1N1 influenza virus emerged in Mexico and quickly spread to other countries, including China. According to the WHO statistics, the virus has killed more than 18000 people. Influenza A virus belongs to the orthomyxoviridae family along with influenza viruses B and C. The influenza A virion is an enveloped RNA virus of spherical to ovoid shape measuring 80–120 nm in diameter. They contain a single-stranded, negative sense, segmented RNA genome consisting of eight segments of viral RNA (vRNA), which encode 11 known proteins [4]. The non-structural protein 1 (NS1) is the most important viral regulatory factor during infection. It is translated from a transcript of the segment eight and plays various roles in regulating viral replication mechanisms, host innate/adaptive immune responses, and cellular signalling pathways. All of these functions of NS1 rely on its ability to participate in a multitude of protein-protein and protein-RNA interactions [5]. To date, over twenty cellular factors have been described which interact with NS1. These include retinoic acid-inducible gene I (RIG-I) [6], poly(A)-binding protein I (PABPI) [7], p85beta [8], importin-α, nucleolin [9], NS1-Binding protein (NS1-BP) [10], eukaryotic initiation factor 4GI (eIF4GI) [7], hStaufen [11], NS1-I [12], protein kinase R (PKR) [13], PKR activator (PACT), the 30-kDa subunit of the cleavage and polyadenylation specificity factor (CPSF30) [14], poly(A)-binding protein II (PABPII) [15], cellular adaptor protein Crk/CrkL [16], PDZ domain-containing proteins [5], the viral polymerase, and components of the cellular mRNA nuclear export machinery (E1B-Adaptor Protein 5 (E1B-AP5), p15, nuclear export factor 1 (NXF1), and nuclear/cytoplasm shuttling factor Rae1) [17] However, in view of the extreme multifunctional nature of NS1, more cellular factors maybe need to associate to this protein so as to fulfill different functions. It is reasonable to assume there are other unidentified interaction partners of NS1 protein.

Despite our substantial knowledge of this amazing and fascinating protein, much still remains to be learnt of its roles in the virus replication cycle. To gain further insight into the role of NS1, we made efforts to utilize influenza Virus A/Beijing/501/2009(H1N1) NS1 to find novel cellular factors that interact with NS1. To this end, a tandem affinity purification (TAP) system was chosen for this study. The key feature of TAP system is the use of two different affinity purification tags, they have gentle washing and elution conditions that allow the protein–protein interactions to remain intact, this not only allow for isolation of exceptionally clean proteins without disrupting the targeted complex, but increase the amount of the resulting purified protein complex. Moreover, as the histopathological and virological finding in fatal cases of 2009 H1N1 revealed that the 2009 H1N1 virus infected type II pneumocytes and caused diffuse alveolar damage (DAD), and potential infection in alveolar epithelial cells is also the main feature that differentiates it from seasonal influenza strains [18], [19]. Human lung adenocarcinoma cell line A549 was used. By use of these, we identified a cellular factor, β-tubulin, as new interaction partner of NS1 protein. In addition, the disruption of the microtubule network and apoptosis were also observed on NS1-transfected A549 cells. Our finding suggested that NS1 affects cellular functions through interaction with β-Tubulin.

**Figure 1:**
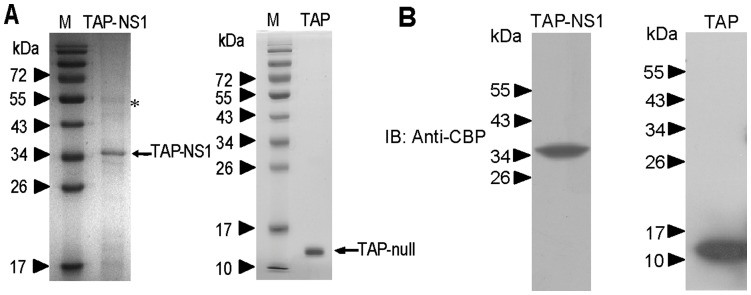
Purification of cellular interaction partner of influenza strains A/Beijing/501/2009(H1N1) NS1. A, The purified protein complexes were detected by SDS-PAGE. Left panel: the protein complexes purified from A549 cells transfected with pnTAP-NS1 plasmids. Right panel: the protein complexes purified from A549 cells transfected with pnTAP vector. M represents protein marker. The bait protein bands and its cellular interaction proteins identified by mass spectrometry are indicated by an arrow and an asterisk, respectively. B, The purified protein complexes were detected by immunoblotting with antibodies against CBP. Left panel: the protein complexes purified from A549 cells transfected with pnTAP-NS1 plasmids. Right panel: the protein complexes purified from A549 cells transfected with pnTAP vector.

**Figure 2:**
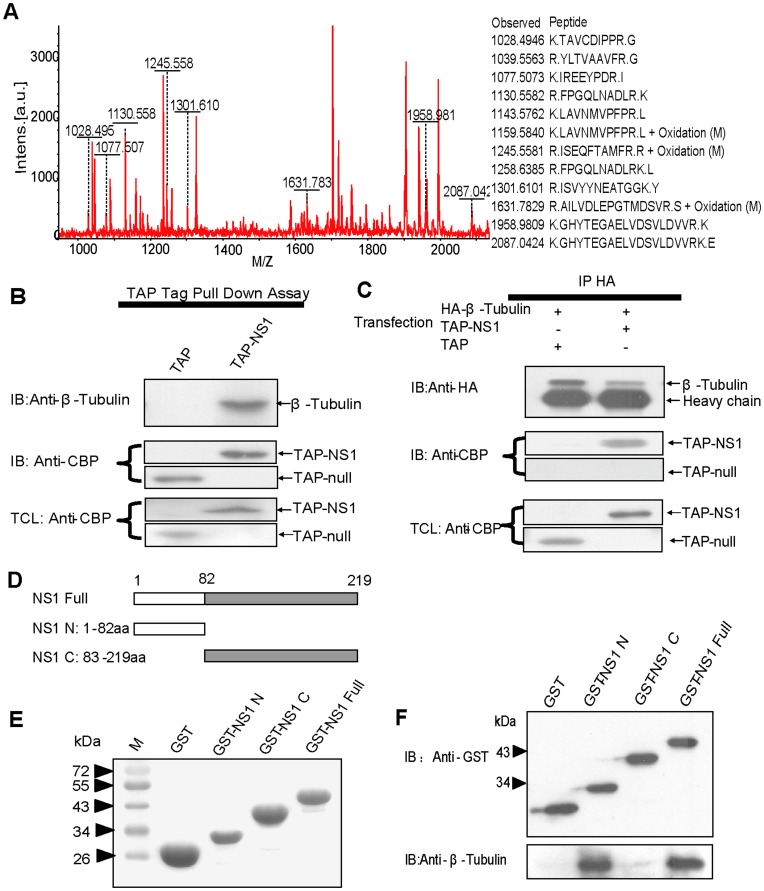
Identification of β-tubulin as a novel NS1-binding protein. (A) Peptide mass fingerprinting of the 55 kDa protein. The protein was identified as β-tubulin using a program, MASCOT, and the peptides assigned to those of β-tubulin are shown. (B) Confirmation of the 55 kDa protein band in TAP purified protein complexes as β-tubulin. The protein complexes purified from A549 cells transfected with pnTAP-NS1 plasmids and that from pnTAP transfected cells were immunoblotted with the anti-β-tubulin antibody (top panel) or anti-CBP antibody (middle panel). The total cell lysate was also immunoblotted with the anti-CBP antibody (bottom panel). (C) Co-immunoprecipitation analysis of β-tubulin and NS1. The precipitates obtained were immunoblotted with the anti-β-tubulin antibody (top panel) or anti-CBP antibody (middle panel). The total cell lysate was immunoblotted with the anti-CBP antibody (bottom panel). (D) An illustration of the various NS1 truncations used to map the β-tubulin-binding domain in the NS1 protein. NS1 Full (NS1 full-length); NS1 N (NS1 N- terminal domain, that is the RNA binding domain); and NS1 C (NS C-terminal domain, that is the effector domain). (E–F) The N-terminal domain of NS1 interacts with β-tubulin. Left panel: the purified GST-fused NS1 proteins stained by coomassie brilliant blue. Right panel: the purified GST-fused NS1 proteins complexes obtained were detected by immunoblotting with anti-GST antibodies (top panel) or anti-β-tubulin antibodies (bottom panel).

## Materials and Methods

### Cell and the Viral Total RNA

A549 (ATCC CCL-185) cells were grown in Dulbecco’s modified Eagle’s medium (DMEM) (HyClone, USA) supplemented with 10% fetal bovine serum (FBS, GIBCO, USA), 100 IU penicillin and 100 µg/ml streptomycin (HyClone, USA). The total RNA of influenza strains A/Beijing/501/2009(H1N1) was kindly provided by Dr. Bohua Liu (Department of virology, Beijing Institute of Microbiology and Epidemiology).

**Figure 3:**
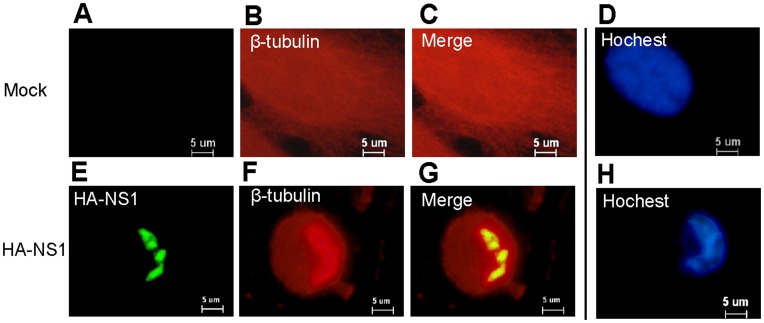
Co-localization of NS1 and β-tubulin in the nucleus, and Influenza virus A/Beijing/501/2009(H1N1) NS1 induce apoptosis. (A), (B), (C), (E), (F), (G). A549 cells were transfected with pCMV5-HA-NS1 and control vector pCMV5, respectively. NS1 was apparent from 24 h post-transfection, mainly in nucleus of A549 cells transfected with pCMV5-HA-NS1 (green color) (Figure 3E). On the other hand, β-tubulin was stained in nucleus and cytoplasm (red color) (Figure 3F).The signals of NS1 and β-tubulin clearly overlapped in nucleus (Figure 3G). (D), (H). The A549 cells transfected with pCMV5-HA-NS1 were stained with Hoechst 33342, and exhibited a stronger blue fluorescence and condensated and fragmented nuclear at 24 h post transfection (Figure 3H).

### Plasmid Constructions

The NS1 cDNA was amplified by RT-PCR using primers 5′-CGC GGATCC ATG GAC TCC AAC ACC ATG TCA AGC T-3′ (BamHI site, underlined) and 5′-CCG GAATTC TCA TTT CTG CTC TGG AGG TAG TGA A-3′ (EcoRI site, underlined) and were ligated between the BamHI and EcoRI sites of pnTAP vector (Stratagene); The correct sequence of all constructs described above was verified by sequencing.

### NS1-TAP Expression and Purification of the Protein Complexes

Ten T175-cm^2^ cell culture flasks of 90% confluence A549 cells were transfected with pnTAP-NS1 plasmids by using Lipofectamine™ 2000 Reagent (Invitrogen) according to the manufacturer’s protocol. In parallel, A549 cells were transfected with pnTAP vector as control. Approximately 48 hours post-transfection, the cells were washed three times with PBS, then 5 ml of ice cold PBS was added to each flask to prepare the cell suspensions, the cells were harvested by centrifuging for 10 minutes at 1500 × g. After removing the PBS, the protein complexes were purified by using InterPlay TAP Purification Kit (Stratagene, catalog #240107) according to the manufacturer’s instructions. To detect the purified proteins, the protein preparation were resolved on 15% SDS-PAGE gels and stained with Coomassie Blue solution.

### Peptide Mass Fingerprinting Analysis

To characterize the TAP-purified protein, the protein bands were excised from the Coomassie Blue-stained SDS-PAGE gel, in-gel digested by trypsin, and analyzed by MALDI-TOF mass spectrometer AXIMA-QIT (Beijing Genomics Institute). Proteins were identified from peptide fragments by comparison to theoretical digests of the human proteome using MASCOT search tools.

### Co-immunoprecipitation Analysis

To exclude the possibility that the interacting partner might represent unspecific factor, and further confirm the specific interaction, co-immunoprecipitation experiments were performed. The β-tubulin cDNA was amplified by RT-PCR using primers 5′-GGA ATTC CATATG ATG AGG GAA ATC GTG CAC ATC CAG G-3′ (NdeI site, underlined) and 5′-TCC CCCGGG TTA GGC CTC CTC TTC GGC CTC CTC-3′ (SmaI site, underlined). After being digested with NdeI and SmaI, the cDNA fragment was cloned into a NdeI-SmaI digested pCMV5-HA vector. A549 cells were co-transfected with expression plasmids pnTAP-NS1 and pCMV5-HA-β-tubulin or pnTAP vector and pCMV5-HA-β-tubulin. Approximately 24 hours post-transfection, the cells were harvested after washing with PBS, and then resuspended inA549 cells were lysed in lysis buffer A (20 mM Tris-HCl (pH7.4), 150 mM NaCl, 1 mM EDTA, 1 mM EGTA, 1% Trion X-100, 2.5 mM sodium pyrophosphate, 1 mM β-glycerol phosphate, 1 mM sodium orthovandate, 10 µl/ml Protease Inhibitor Cocktail (Sigma) and 1 mM PMSF). After centrifugation at 10000 g for 15 min, the supernatant was collected and used as whole cell extract for later co-immunoprecipitation analysis. By using protein A/G beads (Santa Cruz Biotechnology) in the presence of antibody against HA (Santa Cruz Biotechnology), co-immunoprecipitation experiment was performed as described previously [20]. The co-immuoprecipitated products obtained were then subjected to SDS-PAGE and western blot analysis.

### Immunofluorescence Staining

A549 cells were transfected with the expression plasmids pCMV5-HA-NS1 and control vector pCMV5 in suspension by using Lipofectamine 2000 (Invitrogen) respectively, then plated in a 24 well dish containing glass cover slips. Cells were fixed with 4% paraformaldehyde in PBS for 1 h, washed once with PBS and permeablized with 0.2% Triton in PBS for 30 min. After washing with PBS, cells were incubated with 10% goat serum in PBS for 1 h at 37°C to block unspecific binding of antibodies. Next, the primary antibody-mix (polyclonal anti-HA 1∶100; monoclonal anti-β-tubulin 1∶100 (Santa Cruz Biotechnology) in PBS containing 1% BSA was added for 1 h at RT. Cells were washed three times with PBS before the fluorescence-labeled secondary antibody mixture (FITC linked anti-mouse IgG 1∶200 and TRITC linked anti-rabbit IgG 1∶200 in PBS containing 1% BSA) was added for 1 h at 37°C. After cells were washed three times with PBS and rinsed with water, the cover slips were mounted with Fluorescent Mounting Medium on slides and observed with OLYMPUS IX81 microscope.

A cell that is undergoing apoptosis demonstrates nuclear condensation and DNA fragmentation, which can be detected by staining with Hoechst 33342 and fluorescence microscopy [21]. To determine whether apoptosis was induced in the A549 cells transfected with influenza virus A/Beijing/501/2009(H1N1) NS1, coverslips with adherent transfected A549 cells were collected at 24 h post transfection and washed, and the A549 cells were fixed with 4% paraformaldehyde and stained with Hoechst 33342 (100 ng/ml, Sigma, USA) for 20 min at room temperature. The coverslips were washed, mounted on glass slides, and stored at 4°C until quantification by fluorescence microscopy could be performed.

### Mapping of the β-tubulin-binding Domain in the NS1 Protein

NS1 is notionally divided into two distinct functional domains: an N-terminal RNA-binding domain and a C-terminal ‘effector’ domain. To determine which domain of NS1 binds with β-tubulin, GST-fused NS1 constructs were prepared. NS1 fragments were amplified using the following primer sets: for full-length NS1 (NS1full, amino acids 1-219), 5′-CGC GGATCC ATG GAC TCC AAC ACC ATG TCA AGC T-3′ and 5′-TCC CCCGGG AA TCA TTT CTG CTC TGG AGG TAG TGA AGG-3′; for the RBD of NS1 (NS1 N, amino acids 1–81), 5′-CGC GGATCC ATG GAC TCC AAC ACC ATG TCA AGC T-3′ and 5′- TCC CCCGGG CTA TGC AAT TGT CAT TCT AAG TGT CTC G-3′; and for the ED of NS1 (NS1 C, amino acids 82–219), 5′-CGC GGATCC TCT GTA CCT ACT TCG CGC TAC CTT T-3′ and 5′-TCC CCCGGG AA TCA TTT CTG CTC TGG AGG TAG TGA AGG-3′; Each NS1 fragment was digested with BamHI and SmaI and cloned into BamHI-SmaI digested pGEX-4T1 vector. The proteins were induced by 0.2 mM IPTG overnight at 16°C in LB and purified with glutathione Sepharose beads (GE Healthcare), free glutathione was removed by dialysis.

A549 cells were lysed with buffer A for 30 min on ice and then centrifuged at 16000 × g for 30 min at 4°C. The supernatant was collected and used as the total cell lysate. Each GST-fused NS1 was added into the total cell lysate, then the mixture was incubated with glutathione Sepharose beads for 1 h at 4°C. After extensive washing, the bound materials were eluted with 20 mM glutathione in 50 mM Tris-Cl pH 8.0. The eluate was resolved on SDS-PAGE (14% gel) and analyzed by immunoblotting using anti-β-tubulin (Santa Cruz Biotechnology).

## Results

### β-tubulin was Pulled Down Together with NS1 Using the N-terminal TAP Affinity Tags and Identified as a Novel NS1-binding Protein

The representative result of the purified protein complexes is shown in **Figure 1A**. As confirmed by western blot, NS1 and interacting partners were major in the soluble portion of whole cell extract (**Figure 1B**). When the pattern of protein bands was compared between TAP-NS1 complexes and TAP-Null complexes, one major band (about 55 kDa, indicated by asterisk) was specific to the TAP-NS1 complexes on Coomassie Blue-stained gel. Although there are some other minor bands, we focus on the 55 kDa band. To identify this protein, the observed 55 kDa band was analyzed by using a MALDI-TOF mass spectrometer. The peptide mass fingerprinting pattern of the band was obtained successfully and applied to query a database search engine, MASCOT. The best match of the 55 kDa protein was with β-tubulin, a multifunctional cytoskeletal protein (**Figure 2A**). Overall, 12 peptides, ranging in size from 8 to 19 amino acids, were matched, representing a 33% sequence coverage of β-tubulin (141 of a total of 426 amino acids). By immunoblotting using an anti-β-tubulin monoclonal antibody, we confirmed that the detected 55 kDa band was β-tubulin in the TAP-NS1 complexes (**Figure 2B**). β-tubulin was not detected in parallel analysis with the TAP-Null complexes (mock) (**Figure 2B**).

The association of β-tubulin and NS1 in A549 cell lysate was further confirmed by conventional co-immunoprecipitation using an anti-HA rabbit polyclonal antibody. As expected, TAP-NS1 was detected in the precipitates of A549 cells co-tranfected with pnTAP-NS1 and pCMV5-HA-β-tubulin by the anti-calmodulin binding peptide (CBP) antibody (**Figure 2C**), whereas in control co-immunoprecipitation using pnTAP vector and pCMV5-HA-β-tubulin co-transfected cells, no TAP-NULL was detectable.

### The N terminal Domain of NS1 is Responsible for Binding with β-tubulin

Among the three fragments of influenza virus A/Beijing/501/2009(H1N1) NS1, as seen in the results **Figure 2D–F**, β-tubulin was pulled down with GST-NS1 full or GST-NS1 N but not with GST-NS1 C (**Figure 2F**).

### Co-localization of NS1 and β-tubulin in Cells

A549 cells were transfected with pCMV5-HA-NS1, NS1 was apparent from 24 h post-transfection, mainly in nucleus (green color) (**Figure 3E**). On the other hand, β-tubulin was stained in nucleus and cytoplasm (red color) (**Figure 3F**).The signals of NS1 and β-tubulin clearly overlapped in nucleus (**Figure 3G**).

### Apoptotic and Cytoskeleton Cell Morphology Observation

A549 cells were transfected with influenza virus A/Beijing/501/2009(H1N1) NS1 for 24 h and stained with Hoechst 33342. As illustrated in in **Figure 3D and 3H**, the tranfected cells exhibited a stronger blue fluorescence and condensated and fragmented nuclear after expression NS1 for 24 hours.

In addition, an intriguing phenomenon was observed in immunofluorescence staining test. Transfecting A549 cells with plasmid pCMV5-HA-NS1 induced visible changes in microtubule structure in accordance with the disassembly of tubulin polymers at 24 h post transfection (**Figure 3F**), whereas mock transfection of A549 cells with pCMV5 vector did not induce perceptible changes in microtubule structure.

## Discussion

The β-tubulin is the main constituent of *microtubules (MTs)*, *MTs* are dynamic, polarized polymers composed of α/β-tubulin heterodimers, and ubiquitous cytoskeleton components that play a key role in various cellular processes relating to cell shape and division, motility, and intracellular trafficking [22], [23]. *MTs* have important functions in the life cycle of most viruses [24], [25]. In the present study, we identified β-tubulin as a novel interaction partner of influenza A virus NS1 protein, the two proteins co-localize in the nucleus of A549 cell transfected with NS1. As β*-tubulin was generally regarded as a cytosolic protein,* only β(α)-tubulin was found be present in few normal cells *and* a variety of cancerous cell lines [26], [27]. *Therefore we presumed it should be* β(α)-tubulin *which interacts with NS1 in A549 cells.* NS1 consists of two functional domains, the C-terminal effector domain and the N-terminal RNA-binding domain. Here we determined that the RNA-binding domain of NS1 is responsible for binding with the β-tubulin. In addition, we also observed the depolymerization of the MT network on NS1-transfected human A549 Cells. For many anticancer compounds such as taxanes, isochaihulactone and the Vinca alkaloids, interfere with tubulin polymerization and microtubule depolymerization by binding to β-tubulin, and there is no evidence that interaction of NS1 with other known cellular factors induce depolymerization of MT on cells, therefore we assume that the interaction influenza virus A/Beijing/501/2009(H1N1) NS1 with β-tubulin induces disruption of the MT network on NS1-transfected human A549 Cells.

Apoptosis plays an important role in the pathogenesis of many infectious diseases, including those caused by viruses [28], [29]. Influenza viruses have been reported to induce apoptosis in numerous cell types, both in vivo [30], [31] and in vitro [32]. Several viral proteins (M1, NS1, and PB1-F2) from different strains of human influenza viruses have been shown to induce or inhibit apoptosis in human cells [33], [34], [35]. Ning Yang et al. (2011) recently reported that the 2009 pandemic H1N1 strain, A/Wenshan/01/2009, induce apoptotic cell death in epithelial cells of the human respiratory tract [32]. Our results indicated that *influenza virus* A/Beijing/501/2009(H1N1) *NS1 alone can induce apoptosis on A549 cells.* As the two isolates have the same origin, it is not clear whether NS1 play key role on apoptosis induced by influenza virus A/Wenshan/01/2009. *S*everal cell signaling pathways have been showed to be involved in the cell death process [36], [37], [38]. Though the exact signaling pathway that *influenza virus* A/Beijing/501/2009(H1N1) *NS1 induce apoptosis on A549 cells is not clear, progress made in the mechanism that* microtubule depolymerization agents *activate apoptosis may provides some helpful information.* Previous studies have showed that microtubule depolymerization agents interfere with tubulin polymerization and microtubule depolymerization, thereby arrest the cell cycle in G2/M phase and disrupt normal cell division, further act through several types of kinases, leading to phosphorylation cascades and the activation of cyclin B1/cdc2 complex and Bcl-2 phosphorylation, finally initiates the apoptotic cascade [39], [40], [41]. *Our observation indicated influenza virus* A/Beijing/501/2009(H1N1) *NS1 caused* G2-M cell cycle arrest *(data not shown), moreover c*aspase 3-dependent apoptosis was showed to be involved in the homologous strain A/Wenshan H1N1-induced A549 cell and CNE-2Z cell death. Taken together, we presumed that the interaction of *influenza virus* A/Beijing/501/2009(H1N1) *NS1 with* β-tubulin depolymerized MT network and thereby disrupt normal cell division and commit the cell to apoptosis, thereby facilitate virus replication and indirectly contribute to virus pathogenicity. However, the exact role of NS1 on apoptosis induced by the 2009 pandemic H1N1 virus needs further investigation.

In summary, the present study provides evidence that β-tubulin represent a novel interaction partner of influenza A virus NS1 protein. The RNA-binding domain of NS1 is responsible for binding with β-tubulin. The interaction of NS1 with β-tubulin disrupts the cellular microtubule network and induces apoptosis on human A549 cells.

## References

[1] Wright PF WR (2001) Orthomyxoviruses. In: Fields Virology fourth edition, Knipe DM, Howley PM eds, Lippincott, Philadelphia. 1533–1579.

[2] Zimmer SM, Burke DS (2009) Historical perspective–Emergence of influenza A (H1N1) viruses. N Engl J Med 361: 279–285. 19564632 10.1056/NEJMra0904322

[3] Simonsen L, Clarke MJ, Williamson GD, Stroup DF, Arden NH, et al (1997) The impact of influenza epidemics on mortality: introducing a severity index. Am J Public Health 87: 1944–1950. 9431281 10.2105/ajph.87.12.1944 PMC1381234

[4] Palese P, Show ML (2007) Orthomyxoviridae: the viruses and their replication. In: Knipe DM, Howley PM, editors. Fields Virology. Philadelphia: Lippincott Williams & Wilkins. 1647–1689.

[5] Hale BG, Randall RE, Ortin J, Jackson D (2008a) The multifunctional NS1 protein of influenza A viruses. J Gen Virol 89: 2359–2376. 18796704 10.1099/vir.0.2008/004606-0

[6] Mibayashi M, Martinez-Sobrido L, Loo YM, Cardenas WB, Gale MJ, et al (2007) Inhibition of retinoic acid-inducible gene I-mediated induction of beta interferon by the NS1 protein of influenza A virus. J Virol 81: 514–524. 17079289 10.1128/JVI.01265-06 PMC1797471

[7] Burgui I, Aragon T, Ortin J, Nieto A (2003) PABP1 and eIF4GI associate with influenza virus NS1 protein in viral mRNA translation initiation complexes. J Gen Virol 84: 3263–3274. 14645908 10.1099/vir.0.19487-0

[8] Hale BG, Jackson D, Chen YH, Lamb RA, Randall RE (2006) Influenza A virus NS1 protein binds p85beta and activates phosphatidylinositol-3-kinase signaling. Proc Natl Acad Sci U S A 103: 14194–14199. 16963558 10.1073/pnas.0606109103 PMC1599933

[9] Murayama R, Harada Y, Shibata T, Kuroda K, Hayakawa S, et al (2007) Influenza A virus non-structural protein 1 (NS1) interacts with cellular multifunctional protein nucleolin during infection. Biochem Biophys Res Commun 362: 880–885. 17767916 10.1016/j.bbrc.2007.08.091

[10] Wolff T, O’Neill RE, Palese P (1998) NS1-Binding protein (NS1-BP): a novel human protein that interacts with the influenza A virus nonstructural NS1 protein is relocalized in the nuclei of infected cells. J Virol 72: 7170–7180. 9696811 10.1128/jvi.72.9.7170-7180.1998 PMC109939

[11] Falcon AM, Fortes P, Marion RM, Beloso A, Ortin J (1999) Interaction of influenza virus NS1 protein and the human homologue of Staufen in vivo and in vitro. Nucleic Acids Res 27: 2241–2247. 10325410 10.1093/nar/27.11.2241 PMC148787

[12] Wolff T, O’Neill RE, Palese P (1996) Interaction cloning of NS1-I, a human protein that binds to the nonstructural NS1 proteins of influenza A and B viruses. J Virol 70: 5363–5372. 8764047 10.1128/jvi.70.8.5363-5372.1996 PMC190494

[13] Tan SL, Katze MG (1998) Biochemical and genetic evidence for complex formation between the influenza A virus NS1 protein and the interferon-induced PKR protein kinase. J Interferon Cytokine Res 18: 757–766. 9781815 10.1089/jir.1998.18.757

[14] Twu KY, Noah DL, Rao P, Kuo RL, Krug RM (2006) The CPSF30 binding site on the NS1A protein of influenza A virus is a potential antiviral target. J Virol 80: 3957–3965. 16571812 10.1128/JVI.80.8.3957-3965.2006 PMC1440456

[15] Chen Z, Li Y, Krug RM (1999) Influenza A virus NS1 protein targets poly(A)-binding protein II of the cellular 3′-end processing machinery. EMBO J 18: 2273–2283. 10205180 10.1093/emboj/18.8.2273 PMC1171310

[16] Heikkinen LS, Kazlauskas A, Melen K, Wagner R, Ziegler T, et al (2008) Avian and 1918 Spanish influenza a virus NS1 proteins bind to Crk/CrkL Src homology 3 domains to activate host cell signaling. J Biol Chem 283: 5719–5727. 18165234 10.1074/jbc.M707195200

[17] Qiu Y, Krug RM (1994) The influenza virus NS1 protein is a poly(A)-binding protein that inhibits nuclear export of mRNAs containing poly(A). J Virol 68: 2425–2432. 7908060 10.1128/jvi.68.4.2425-2432.1994 PMC236720

[18] Rosen DG, Lopez AE, Anzalone ML, Wolf DA, Derrick SM, et al (2010) Postmortem findings in eight cases of influenza A/H1N1. Mod Pathol 23: 1449–1457. 20802471 10.1038/modpathol.2010.148

[19] Nakajima N, Sato Y, Katano H, Hasegawa H, Kumasaka T, et al (2012) Histopathological and immunohistochemical findings of 20 autopsy cases with 2009 H1N1 virus infection. Mod Pathol 25: 1–13. 21874012 10.1038/modpathol.2011.125

[20] Rui HL, Fan E, Zhou HM, Xu Z, Zhang Y, et al (2002) SUMO-1 modification of the C-terminal KVEKVD of Axin is required for JNK activation but has no effect on Wnt signaling. J Biol Chem 277: 42981–42986. 12223491 10.1074/jbc.M208099200

[21] Allen S, Sotos J, Sylte MJ, Czuprynski CJ (2001) Use of Hoechst 33342 staining to detect apoptotic changes in bovine mononuclear phagocytes infected with Mycobacterium avium subsp. paratuberculosis. Clin Diagn Lab Immunol 8: 460–464. 11238240 10.1128/CDLI.8.2.460-464.2001 PMC96081

[22] Nogales E (2000) Structural insights into microtubule function. Annu Rev Biochem 69: 277–302. 10966460 10.1146/annurev.biochem.69.1.277

[23] Heald R, Nogales E (2002) Microtubule dynamics. J Cell Sci 115: 3–4. 11801717 10.1242/jcs.115.1.3

[24] Dohner K, Nagel CH, Sodeik B (2005) Viral stop-and-go along microtubules: taking a ride with dynein and kinesins. Trends Microbiol 13: 320–327. 15950476 10.1016/j.tim.2005.05.010

[25] Greber UF, Way M (2006) A superhighway to virus infection. Cell 124: 741–754. 16497585 10.1016/j.cell.2006.02.018

[26] Walss C, Kreisberg JI, Luduena RF (1999) Presence of the betaII isotype of tubulin in the nuclei of cultured mesangial cells from rat kidney. Cell Motil Cytoskeleton 42: 274–284. 10223634 10.1002/(SICI)1097-0169(1999)42:4<274::AID-CM2>3.0.CO;2-5

[27] Yeh IT, Luduena RF (2004) The betaII isotype of tubulin is present in the cell nuclei of a variety of cancers. Cell Motil Cytoskeleton 57: 96–106. 14691949 10.1002/cm.10157

[28] Young LS, Dawson CW, Eliopoulos AG (1997) Viruses and apoptosis. Br Med Bull 53: 509–521. 9374034 10.1093/oxfordjournals.bmb.a011627

[29] Ludwig S, Pleschka S, Wolff T (1999) A fatal relationship–influenza virus interactions with the host cell. Viral Immunol 12: 175–196. 10532647 10.1089/vim.1999.12.175

[30] Mori I, Komatsu T, Takeuchi K, Nakakuki K, Sudo M, et al (1995) In vivo induction of apoptosis by influenza virus. J Gen Virol 76 (Pt 11): 2869–2873. 10.1099/0022-1317-76-11-2869 7595397

[31] Roulston A, Marcellus RC, Branton PE (1999) Viruses and apoptosis. Annu Rev Microbiol 53: 577–628. 10547702 10.1146/annurev.micro.53.1.577

[32] Yang N, Hong X, Yang P, Ju X, Wang Y, et al (2011) The 2009 pandemic A/Wenshan/01/2009 H1N1 induces apoptotic cell death in human airway epithelial cells. J Mol Cell Biol 3: 221–229. 21816972 10.1093/jmcb/mjr017

[33] Chanturiya AN, Basanez G, Schubert U, Henklein P, Yewdell JW, et al (2004) PB1-F2, an influenza A virus-encoded proapoptotic mitochondrial protein, creates variably sized pores in planar lipid membranes. J Virol 78: 6304–6312. 15163724 10.1128/JVI.78.12.6304-6312.2004 PMC416516

[34] Stasakova J, Ferko B, Kittel C, Sereinig S, Romanova J, et al (2005) Influenza A mutant viruses with altered NS1 protein function provoke caspase-1 activation in primary human macrophages, resulting in fast apoptosis and release of high levels of interleukins 1beta and 18. J Gen Virol 86: 185–195. 15604446 10.1099/vir.0.80422-0

[35] Zhirnov OP, Ksenofontov AL, Kuzmina SG, Klenk HD (2002a) Interaction of influenza A virus M1 matrix protein with caspases. Biochemistry (Mosc) 67: 534–539. 12059772 10.1023/a:1015542110798

[36] Ehrhardt C, Wolff T, Pleschka S, Planz O, Beermann W, et al (2007) Influenza A virus NS1 protein activates the PI3K/Akt pathway to mediate antiapoptotic signaling responses. J Virol 81: 3058–3067. 17229704 10.1128/JVI.02082-06 PMC1866065

[37] McLean JE, Datan E, Matassov D, Zakeri ZF (2009) Lack of Bax prevents influenza A virus-induced apoptosis and causes diminished viral replication. J Virol 83: 8233–8246. 19494020 10.1128/JVI.02672-08 PMC2715773

[38] Nencioni L, De Chiara G, Sgarbanti R, Amatore D, Aquilano K, et al (2009) Bcl-2 expression and p38MAPK activity in cells infected with influenza A virus: impact on virally induced apoptosis and viral replication. J Biol Chem 284: 16004–16015. 19336399 10.1074/jbc.M900146200 PMC2708894

[39] Wang LG, Liu XM, Kreis W, Budman DR (1999) The effect of antimicrotubule agents on signal transduction pathways of apoptosis: a review. Cancer Chemother Pharmacol 44: 355–361. 10501907 10.1007/s002800050989

[40] Jordan A, Hadfield JA, Lawrence NJ, McGown AT (1998) Tubulin as a target for anticancer drugs: agents which interact with the mitotic spindle. Med Res Rev 18: 259–296. 9664292 10.1002/(sici)1098-1128(199807)18:4<259::aid-med3>3.0.co;2-u

[41] Liao CH, Pan SL, Guh JH, Chang YL, Pai HC, et al (2005) Antitumor mechanism of evodiamine, a constituent from Chinese herb Evodiae fructus, in human multiple-drug resistant breast cancer NCI/ADR-RES cells in vitro and in vivo. Carcinogenesis 26: 968–975. 15705600 10.1093/carcin/bgi041

